# miR-221/222 sponge abrogates tamoxifen resistance in ER-positive breast cancer cells through restoring the expression of ERα

**DOI:** 10.1186/s43556-021-00045-0

**Published:** 2021-06-30

**Authors:** Yan Xiu Ouyang, Jun Feng, Zun Wang, Guo Jun Zhang, Min Chen

**Affiliations:** 1 Cancer Center & Department of Breast and Thyroid Surgery, Xiang’an Hospital of Xiamen University, School of Medicine, Xiamen University, No. 2000, Xiang’an Road East, Xiamen, 361101 Fujian China; 2 Clinical Central Research Core, Xiang’an Hospital of Xiamen University, No. 2000, Xiang’an Road East, Xiamen, 361101 Fujian China; 3 ChangJiang Scholar’s Laboratory, Shantou University Medical College, Shantou, 515041 China; 4 Department of Breast and Thyroid Surgery, Shenzhen Baoan Women’s and Children’s Hospital, Jinan University, Shenzhen, 518102 China; 5 Cancer Research Center, School of Medicine, Xiamen University, Xiamen, China; 6 Key Laboratory for Endocrine-Related Cancer Precision Medicine of Xiamen, Xiang’an Hospital of Xiamen University, No. 2000, Xiang’an Road East, Xiamen, 361101 Fujian China

**Keywords:** Breast cancer, Tamoxifen resistance, Sponge, miR-221/222, hTERT promoter

## Abstract

Tamoxifen resistance (TamR) prevents ER-positive breast cancer patients from benefitting from endocrine therapy, and miR-221 or miR-222 plays vital roles in inducing TamR. In this study, we designed synthetic sponges to reverse TamR by targeting these two miRs. First, we established a tamoxifen resistant breast cancer cell line (MCF-7^TamR^), we verified the high expressing level of these two miRs in TamR cells. miR-221 or miR-222 inhibitors rendered MCF-7^TamR^ cells responsive to tamoxifen. Next, we designed a miR-221/222 sponge, which contains total 8 multi-antisense binding sites (MBSs) for these two onco-miRs, and inserted it into CMV promoter- or hTERT promoter-driven expressing vectors. After transfected miR-221/222 sponge expressing vectors into MCF-7^TamR^ cells, we identified a strong interaction between miR-221/222 sponge and endogenous miR-221 or miR-222 by RNA pulldown assay. We also found that miR-221/222 sponge restored the expression of ERα and PTEN, arrested cells in G1 phase, and finally resulted in reduced cell growth and cell migration. Notably, miR-221/222 sponge expressing cells abrogates tamoxifen resistance through restoring the expression of ERα, suggesting that miR-221/222 sponge gene therapy especially driven by tumor specific promoter could provide an effective therapeutic approach against TamR in breast cancer.

**Supplementary Information:**

The online version contains supplementary material available at 10.1186/s43556-021-00045-0.

## Introduction

Breast cancer is the most leading cause of cancer-related death in women [1]. Estrogen receptor (ER) is highly expressed in approximately 70% of breast cancers. Tamoxifen, an antagonist of estrogen, is the most commonly used treatment for both premenopausal and postmenopausal ER-positive (ER^+^) breast cancer patients [2, 3]. Unfortunately, about half of these patients have intrinsic or acquired tamoxifen resistance (TamR) [4, 5], which led to tumor recurrence in many of the patients [6]. TamR remains a major clinical impediment to the effective treatment of ER^+^ breast cancer, and there is a critical and urgent need for restoring tamoxifen sensitivity.

Key regulators and signaling events in TamR include the ER itself and its co-regulators, cross-talk between ER and growth factor signaling [5], and loss or mutation of the ER [7]. miRs represent a class of endogenous small (18–24 nucleotides)noncoding RNAs, which target through binding to the imperfect complementary 3′-untranslated region of the mRNA to “fine-tune or control” the gene degradation and translation [8]. More than 60% of all known human protein-coding genes are directly or indirectly regulated by miRs. Altered expression of specific microRNAs (miRs) are associated with tamoxifen resistance [9] and could be used to predict the outcomes and responses to tamoxifen treatment [10]. OncomiRs are gaining extensive interest due to their potential translational application as therapeutic moieties and targets in cancer [11]. miR-221/222 is a miRNA cluster located on chromosome X, and its genome abnormality contributes to the pathogenesis in multiple advanced cancer [12]. They are also “STAR MOLECULES” in TamR [13–16]. Miller et al first demonstrated that miR-221/222 overexpressing confers TamR in breast cancer [13]. There is a negative regulatory loop between miR-221/222 and ERα, and increased miR-221/222 expressing can induce a transition of breast cancer cells from ER-positive to ER-negative [17].

In theory, modulating onco-miR-221/222 expression by natural non-coding RNAs i.e., antisense oligonucleotides or synthetic miRNA sponge may a potential therapeutic strategy for overcoming TamR. Natural non-coding RNAs serve as regulatory sponges to sequester sequence-specific miRNAs [18, 19]. Thus, synthetic miRNA sponges include miRNA binding sites that mimic those found in mRNAs and are complementary to the 3′-untranslated region (3’UTR) of miRNA response elements (MREs) [20]. These binding sites usually are designed to target either a single specific miRNA or several miRNAs by tandem repeats of identical sites. To achieve long-term effective inhibition of miRNA function, miRNA sponge technology has been developed through the generation of plasmids or viral expression vectors, including those that repeat the seed sequences of multiple miRNAs in a single inhibitor. Synthetic miRNA sponge technology has been developed to continuously cause miRNA loss-of-function in cells [21], especially in cancer cells [22, 23]. Moshiri et al developed novel “miR-221 sponge” vectors that induced cell apoptosis and reduced viability in hepatocellular carcinoma cells through inhibiting of the oncogenic miR-221 [24], suggesting that miRs sponge-based therapeutics might be a useful approach against solid tumors with increased onco-miRs, e.g., miR-221/222 sponges against tamoxifen-resistant breast cancer [16].

In the present study, we analyzed the expression of miR-221 and miR-222 in established MCF-7^TamR^ cells. We inserted tandem bulged miRNA binding sites targeting miR-221 and miR-222 into cytomegalovirus (CMV) promoter- and human telomerase reverse transcriptase (hTERT) promoter-driven expression vectors to generate synthetic tumor-specific miR-221/222 sponges. We investigated the roles of these sponges in cellular function and their potential application for TamR in breast cancer.

## Results

### The characteristics of acquired tamoxifen-resistant MCF-7 models

**Fig. 1:**
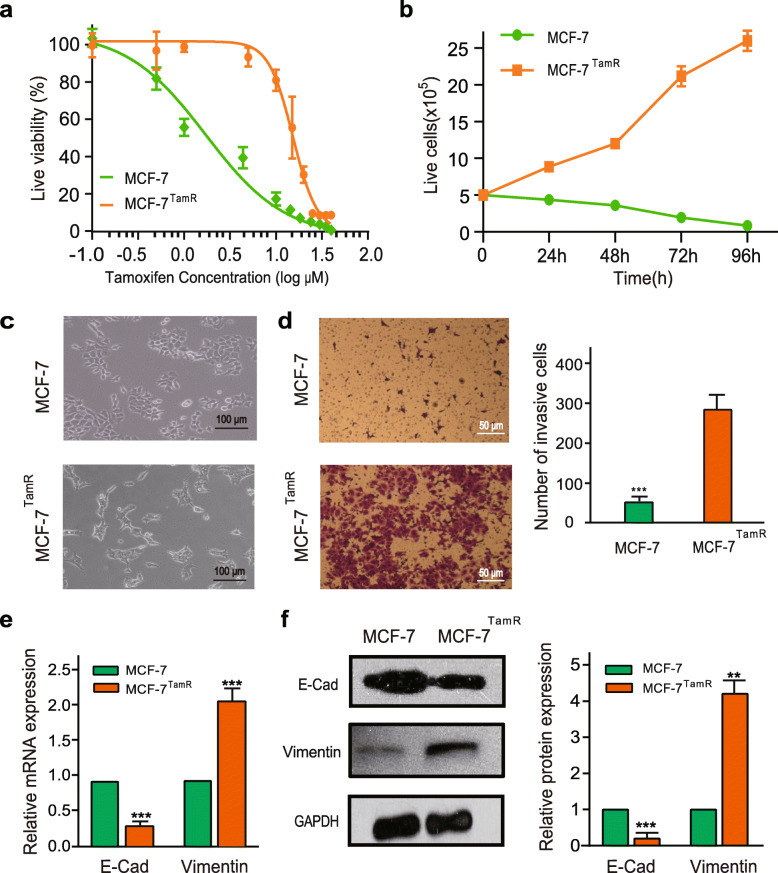
Characterization of tamoxifen-resistant MCF-7 cells. **a** The growth inhibition curves of MCF-7 and MCF-7^TamR^ cells were obtained using the CCK-8 assay following the treatment of 4-hydroxytamoxifen (0–40 μM) for 72 h. **b** The number of live cell of MCF-7 and MCF-7^TamR^ cells was counted during 15 μM 4-hydroxytamoxifen treatment for 4 days. **c** Morphology of MCF-7 and MCF-7^TamR^ cells. **d** MCF-7 and MCF-7^TamR^ cell invasion was determined by the transwell assay (left, morphological comparison of cell penetration; right, quantitative bar graph for the number of migrating cells). Cells were counted in five random fields. Data are presented as the mean ± SD (*n* = 3). ***p < 0.001. **e** The mRNA expression of E-cadherin and Vimentin were determined in MCF-7 and MCF-7^TamR^ cells by qRT-PCR. Data are presented as the mean ± SD (*n* = 3). ***p < 0.001. **f** Western blotting for E-cadherin, Vimentin, and GAPDH in MCF-7 and MCF-7^TamR^ cells (left, representative images; right, quantitative bar graph for band density). **p < 0.01, ***p < 0.001

In this study, MCF-7^TamR^ cell line was obtained by prolonged exposure of MCF-7 cells to 4-hydroxytamoxifen-containing medium and verified by cell viability assays. We found that the IC50 of 4-hydroxytamoxifen was 1.56 μM for the parental MCF-7 cells and 13.24 μM for the MCF-7^TamR^ cells (Fig. 1a). The tamoxifen-resistant cells still displayed considerable viability in the presence of 15 μM 4-hydroxytamoxifen (Fig. 1b). As shown in Fig. 1c, the tamoxifen-resistant cells lost tight cell-cell contacts, grew loosely, and spread as individual cell. Moreover, a significant increase of cell migration was observed (Fig. 1d). Consistently, the expression of the epithelial adhesion protein E-cadherin was downregulated in the MCF-7^TamR^ cells compared to the control cells, whereas the expression of the mesenchymal marker vimentin was upregulated (Fig. 1e, f).

### Anti-miR-221 and anti-miR-222 enhanced the sensitivity of MCF-7^TamR^ cells to tamoxifen

**Fig. 2:**
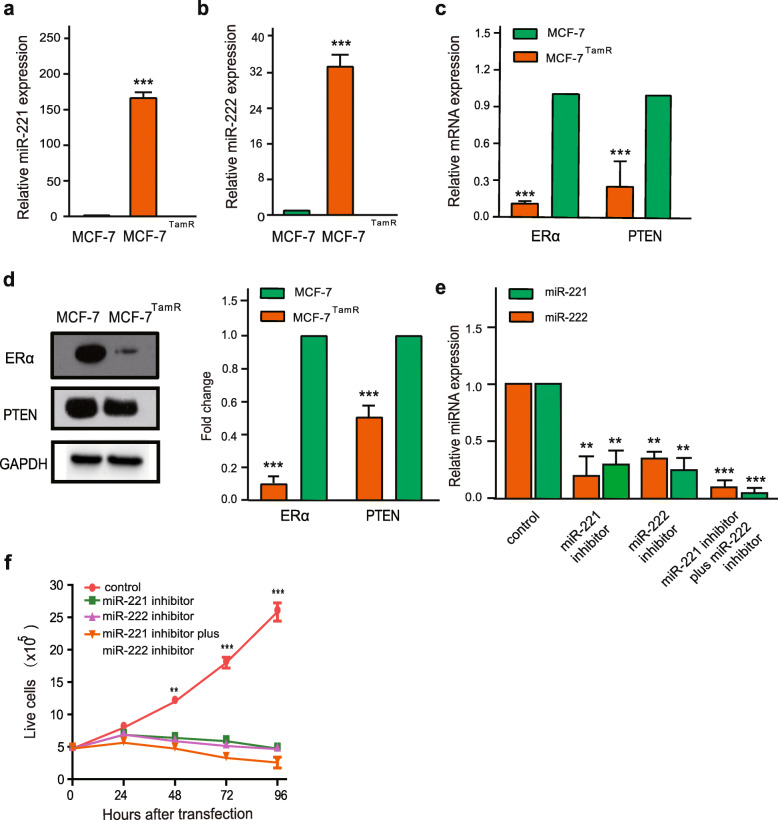
Inhibition of the miR-221/222 network restored the sensitivity of MCF-7^TamR^ cells to tamoxifen. Expression of miR-221 (**a**), miR-222 (**b**), ERα and PTEN (**c**) in MCF-7 and MCF-7^TamR^ cells determined by qRT-PCR. **d** The protein level of ERα and PTEN in MCF-7 and MCF-7^TamR^ cells was analyzed by western blotting. **e** The expression of miR-221 and miR-222 of MCF-7^TamR^ cells was analyzed by qRT-PCR at 48 h after the transfection of miR-221 and miR-222 inhibitors, and **f** The growth of MCF-7^TamR^ cells in the presence of 15 μM 4-hydroxytamoxifen was examined by the CCK-8 assay after the transfection of miR-221 inhibitor, miR-222 inhibitor, miR-221 plus miR-222 inhibitor. Data in a-f were presented as the mean ± SD (*n* = 3). **p < 0.01, ***p < 0.001

The upregulation of miR-221 and miR-222 expression was observed in MCF-7^TamR^ cells compared to the parental MCF-7 cells (Fig. 2a, b). More importantly, the mRNA and protein expression of ERα and PTEN, targets of miR-221/222, were markedly decreased in MCF-7^TamR^ cells (Fig. 2c, d). Subsequently, either silencing of miR-221 or miR-222 alone or in combination inhibited on the growth of MCF-7^TamR^ cells in the presence of 15 μM 4-hydroxytamoxifen (Fig. 2e, f).

### miR-221/222 sponge de-repressed ERα and PTEN expression in MCF-7^TamR^ cells

**Fig. 3:**
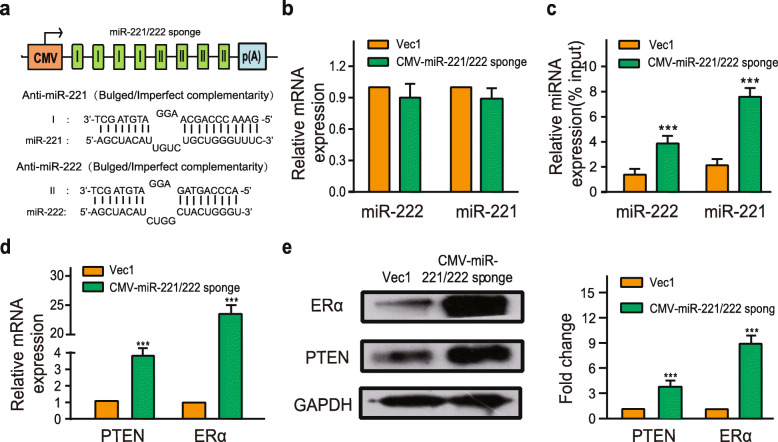
The CMV-miR-221/222 sponge combined miR-221 and miR-222 to de-repress ERα and PTEN expression in MCF-7^TamR^ cells. **a** Schematic of the miR-221/222 sponge expression cassette. I: bulged/imperfect complementarity towards miR-221; II: bulged/imperfect complementarity towards miR-222. **b** The expression of miR-221 and miR-222 was analyzed by qRT-PCR 48 h after the transfection of the CMV-miR-221/222 sponge. Simultaneously, a biotin-labelled miR-221/222 sponge probe was used to pulldown miR-221 and miR-222. Then the amount of miR-221 and miR-222 was assessed by qRT-PCR (**c**). In addition, qRT-PCR and western blotting were used to explore the expression of ERα and PTEN after the transfection of the miR-221/222 sponge expressing vector in MCF-7^TamR^ cells (**d**, **e**). Data in b-e were presented as the mean ± SD (*n* = 3). ***p < 0.001

We designed expression vector containing four miR-221 and four miR-222 binding sites (Fig. 3a). The miR-221/222 sponge contained both miR-221/222 binding sites with an internal mismatch in the middle portion to create a bulge for more potent inhibition of the endogenous cellular miR-221/222. The CMV-miR-221/222 sponges did not change miR-221/222 expression levels (Fig. 3b). Subsequently, RNA pulldown was performed to verify the combination between CMV-miR-221/222 sponge and endogenous miR-221 or miR-222. The pulldown level of miR-221 or miR-222 level was higher in the biotinylated-labeled miR-221/222 sponge than control DNA (Fig. 3c). Additionally, when the vector encoding the CMV-miR-221/222 sponges was transfected into the MCF-7^TamR^ cells, the mRNA and protein expression levels of ERα and PTEN were de-repressed (Fig. 3d, e).

### T-VISA-miR-221/222 sponge significantly suppressed the growth of tamoxifen-resistant cells

**Fig. 4:**
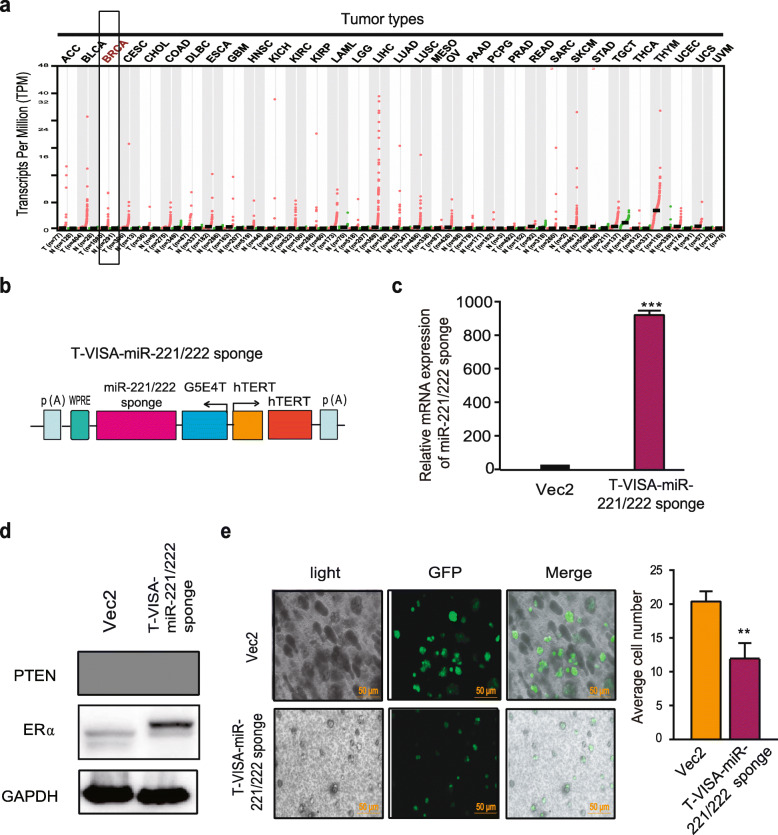
T-VISA -miR-221/222 sponge inhibited cell growth of MCF-7^TamR^ cells in a 3D cell-culture model. **a** hTERT expression profile across all tumor samples and paired normal tissues. Each dot represents the expression in one sample. The red dots represent tumor samples, the green dots represent normal samples. T: Tumor, N: Normal. BRCA: Breast cancer. **b** Schematic representation of the hTERT-based promoter-driven miR-221/222 sponge construct (T-VISA-miR-221/222 sponge). **c** The mRNA expression of the miR-221/222 sponge was analyzed by qRT-PCR after 48 hours transfection with the T-VISA-miR-221/222 sponge expressing vector. **d** The protein level of ERα and PTEN in MCF-7^TamR^ cells was determined by western blotting after 72 hours transfection with the T-VISA-miR-221/222 sponge expressing vector. **e** The effect of the T-VISA-miR-221/222 sponge on cell growth of the GFP-expressing reporter in MCF-7^TamR^ 3D cell culture. The data in **c-e** were presented as the mean ± SD (*n* = 3). **p < 0.01, ***p < 0.001

In order to specifically express miR-221/222 sponge in breast cells, we selected a tumor specific promoter to replace of CMV promoter. Through online database (http://gepia.cancer-pku.cn/), we found that the expression of human telomerase reverse transcriptase mRNA is higher in breast cancer than in normal tissue (Fig. 4a). We constructed a T-VISA-miR-221/222 sponge plasmid (Fig. 4b, c) and analyzed the effects of the T-VISA-miR-221/222 sponge on endogenous PTEN and ERα levels of MCF-7^TamR^ cells. As shown in Fig. 4d, both PTEN and ERα protein levels increased in the MCF-7^TamR^ cells after the transfection of the T-VISA-miR-221/222 sponge. Interestingly, overexpression of the T-VISA-miR-221/222 sponge significantly suppressed the growth of MCF-7^TamR^ cells (Fig. 4e).

### miR-221/222 sponge abrogated TamR through G1 cell cycle arrest and restoration of ERα expression

**Fig. 5:**
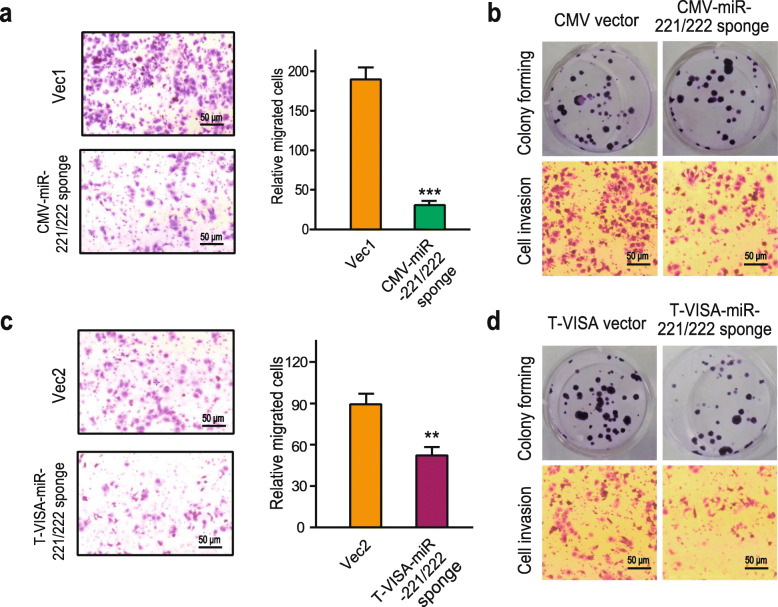
The CMV-miR-221/222 sponge and T-VISA miR-221/222 sponge inhibited tumor progression in MCF-7^TamR^ cells. MCF-7^TamR^ cells were transfected with the miR-221/222 sponge expressing vectors or corresponding control vectors. The effects of the miR-221/222 sponge on cell migration of MCF-7^TamR^ cells were determined by the transwell assay (**a**, **c**), and the colony numbers and the cell invasion of MCF-7TamR cells were determined by cell colony-forming assay and the transwell assay, respectively (**b**, **d**). ***p < 0.001, **p < 0.01

**Fig. 6:**
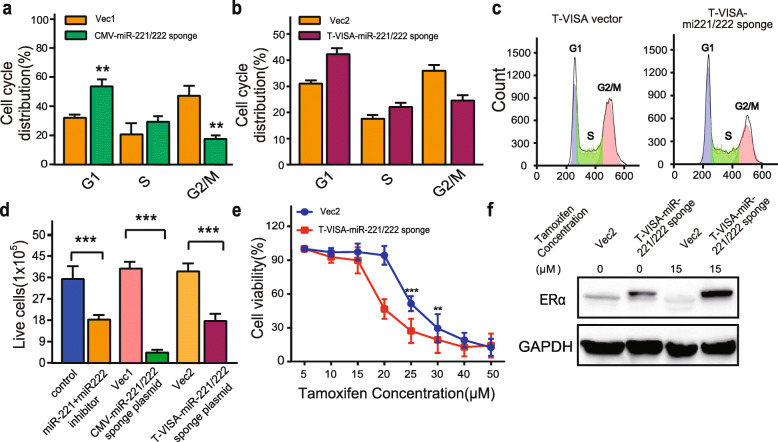
The CMV-miR-221/222 sponge and T-VISA miR-221/222 sponge restored the sensitivity of MCF-7^TamR^ cells to tamoxifen. The effect of sponges on cell cycle was determined by FACS (**a**, **b**, **c**). **d** After transfection with the T-VISA miR-221/222 sponge expressing vector, CMV-miR-221/222 sponge expressing vector or miR-221 plus miR-222 inhibitor, the growth of MCF-7^TamR^ cells in the presence of 15 μM 4-hydroxytamoxifen was determined by the CCK-8 assay. **e** The effect of the T-VISA-miR-221/222 sponge on restoring the sensitivity of MCF-7^TamR^ cells to 4-hydroxytamoxifen (0–40 μM) was determined using the CCK-8 assay. **f** The expression of ERα in MCF-7^TamR^ cells was analyzed by western blotting after 72 hours transfection with the T-VISA-miR-221/222 sponge expressing vector in the presence or absence of 15 μM 4-hydroxytamoxifen. Data in a-e were presented as the mean ± SD (*n* = 3). ***p < 0.001, **p < 0.01

To directly test the effects of the miR-221/222 sponge on cellular function, either the CMV-miR-221/222 sponge or T-VISA-miR-221/222 sponge expressing vectors was transfected into MCF-7^TamR^ cells. There were no visible morphological changes observed after the transfection (Supplementary Figure 1). In Fig. 5a, c, miR-221/222 sponge significantly decreased the migration of MCF-7^TamR^ cells by the transwell assay. Consistent with the changes of cell migration, we observed that miR-221/222 sponge significantly decreased colony numbers, the reduced invasion of MCF-7^TamR^ cells after the transfection with the miR-221/222 sponge expression vector (Fig. 5b, d). Moreover, miR-221/222 sponge increased the proportion of G1 and decreased the proportion of G2/M of the MCF-7^TamR^ cells (Fig. 6a, b, c). Furthermore, miR-221/222 sponge significantly reversed TamR (Fig. 6d, e). In contrast to the control vector transfection, the expression of ERα was restored by miR-221/222 sponge transfection in the absence 4-hydroxytamoxifen, and the restoration level of ERα was even higher in the presence of 15 μM 4-hydroxytamoxifen (Fig. 6f).

## Discussion

Developing sponge technology to silence TamR-related oncomiRs is a rational therapeutic strategy for deciphering the functions of these miRNAs to overcome TamR. In the present study, we confirmed that the inhibition of miR-221/222 restored the sensitivity of MCF-7^TamR^ cells to tamoxifen. Synthetic miR-221/222 sponges as a possible treatment strategy could abrogate tamoxifen resistance in MCF-7^TamR^ cells through upregulation of ERα and PTEN.

Several molecular inhibitors for miRNA, such as antagomiR and miRNA sponge, have been developed and shown promise in the inhibition of cellular function of miRNA. In contrast to antagomiR only inhibiting single complementary miRNA, synthetic miRs sponge contains multi-antisense binding sites (MBSs) for several miRs. Thus, the length of oligonucleotide of synthesized miRs sponge expression cassette was relative long (> 100-mers). Ebert et al were the first to construct Pol II- and Pol III-generated sponges containing MBSs inserted into the 3′ UTR of a destabilized GFP reporter driven by the CMV promoter [25]. Kluiver et al also developed a methodology for the rapid generation of miRNA sponges with up to 20 MBSs [26].

In this study, we designed miR-221/222 sponges that included eight MBSs (4 for miR-221 and 4 for miR-222) to sequester miR-221/222 simultaneously. The CMV promoter has strong activity for sponges expressed in cells; however, it lacks cancer cell specificity. In contrast, the hTERT promoter is much weaker for sponges expressed in cells compared to the CMV promoter, but it is more specific for tumor cells [27, 28]. The versatile targeting vector “VISA” (VP16-GAL4-WPRE integrated systemic amplifier; WPRE, the post-transcriptional regulatory element of the woodchuck hepatitis virus) was invented by Prof. Xie et al, and also verified its strong promoter activity with a prolonged duration of gene expression [29]. In particular, the hTERT promoter-driven T-VISA systems for silencing miRs or overexpressing proapoptotic gene have been successfully applied as therapeutic strategies for cancer in preclinical models [30, 31]. Thus, we investigated the application of hTERT promoter-driven T-VISA systems to silence miR-221 and miR-222 against TamR, and confirmed its ability to restore the expression of miR-221 and miR-222 target genes in MCF-7^TamR^ breast cancer cells. At the same time, the MCF-7^TamR^ cells transfected by hTERT-miR-221/222 sponge T-VISA system restored the sensitivity to tamoxifen treatment.

miRNA inhibitors (i.e., miRNA sponges) can de-repress miRNA targets [25, 32]. miR-221/222 have multiple target genes, including Cip/Kip family members (p21, p27, p57) [13], TIMP3 [12, 33], FOXO3A [34, 35], PUMA [36–38] and PTEN [39], ERα [13, 14, 40] have been implicated in anti-estrogen resistance. In our study, the sensitivity of tamoxifen drug-resistant strains was accompanied with changes in ERα and PTEN. In the present study, we reconfirmed the miR-221/222 sponges de-repressed both ERα and PTEN expression, thereby inducing cell-cycle arrest and suppression of cell growth in 3D-cell culture conditions, which indicated that miR-221/222 sponges might modulate TamR via PTEN and ERα signaling.

In conclusion, the discovery of short non-coding miR-221/222 and our increasing understanding of their functions in TamR, provide potential therapeutic implications for breast cancer. This study is the first to explore synthetic miR-221/222 sponges as molecular therapy to re-sensitize tamoxifen-resistant breast cancer cells to the drug, and this effect may be based on the modulation of ERα and PTEN.

## Materials and methods

### Cell culture

MCF-7 cells were cultured in DMEM (Thermo Fisher Scientific, Inc.) supplemented with 10% fetal bovine serum and 1% penicillin/streptomycin at 37 °C in a humidified incubator with 5% CO_2_.

### Establishment of the MCF-7^TamR^ cell line

The MCF-7^TamR^ cell line was generated as previously described [41]. Briefly, MCF-7 cells were grown continuously in medium containing 1 μM 4-hydroxytamoxifen (Sigma-Aldrich) for 3 months and 3 μM 4-hydroxytamoxifen for at least 9 months.

### Drug sensitivity assay

Cells were incubated with 4-hydroxytamoxifen (0.1 μM, 0.5 μM, 1 μM, 5 μM, 10 μM, 15 μM, 20 μM, 25 μM, 30 μM, 35 μM, 40 μM), and cell proliferation was measured using Cell Counting Kit-8 (CCK-8) (Beyotime Institute of Biotechnology) after different-time treatment. Cell viability were determined by measuring the absorbance at 450 nm using a plate reader.

### Cell survival analysis

Cells were treated with 5 μM 4-hydroxytamoxifen. After different-time treatment, cells were stained with trypan blue and counted by a cell counter (Thermo Fisher Scientific).

### Cell transfection

siRNAs or miRNA sponge expressing vectors were transfected into cells using Lipofectamine 3000 (Life Technology) according to the manufacturer’s instructions. Small interference RNAs (siRNAs) used in this study were purchased from GenePharma (see Supplementary Table 1).

### Western blotting

Protein was separated by SDS-PAGE and transferred onto PVDF membranes. The membranes were incubated with the primary antibodies (see Supplementary Table 2) at 4 °C overnight. After incubated with peroxidase-conjugated goat anti-mouse or anti-rabbit IgG second antibodies, the specific protein band was visualized using super ECL detection reagent (Applygen).

### Construction of the miRNA sponge expressing vectors

The miR-221/222 sponge that contained four copies each of miR-221-3p and miR-222-3p (see Supplementary Table 3) was cloned into the BamHI/Xba I sites of the pLenti-CMV-TO-V5-luc-Puro plasmid (addgene, #w549–1), named as CMV- miR-221/222 sponge expressing vector. Additionally, the T-VISA-miR-221/222 sponge expressing vector was constructed by inserting the miR-221/222 sponge into the Xba I site of the T-VISA plasmid, which was kindly provided by Prof. Xie [30].

### RNA isolation and real-time RT-PCR

According to the manufacturer’s instructions, total mRNA and miRNA were extracted using Trizol reagent (Invitrogen, CA, USA), and cDNA was synthesized from the total RNA using the PrimeScript RT reagent Kit (Takara, Japan). Real-time RT-PCR for gene mRNA expression was performed with SYBR Select Master Mix (Thermo Fisher, MA, USA) and the CFX96 Real-time PCR Detection System (Bio-Rad, CA, USA). Real-time RT-PCR for miRNA expression was carried out using the Hairpin-it miRNAs RT-PCR Quantitation and TaqMan-microRNA Assay kits (GenePharma, Suzhou, China). The primer sequences used for real-time RT-PCR are listed in Supplementary Table 4.

### RNA pulldown

According to the manufacturer’s instructions, the RNA pulldown assay was performed using the μMACS Streptavidin Kit (Miltenyi Biotec Inc). We labelled sponge probe with biotin using transcription and then incubated the probes with an MCF-7 cytoplasmic lysate to form sponge-microRNA complexes. The complexes were combined via chain affinity with magnetic beads and thus separated from other components. After complex elution, we determined by qRT-PCR assays the miR-221/222 cluster that were pulled down. Briefly, total RNA (30 μg) and biotinylated capture DNA (1 μg) were used for pulldown. Then, 300 ng pulldown RNA was analyzed by real-time RT-PCR.

### Colony formation assay

Approximately 1 × 10^3^ MCF-7 cells were cultured incubated in DMEM with 10% FBS at 37 °C. After 14 days, when the colonies were larger than 50 cells, the cells were stained using crystal violet. Each experiment was repeated three time.

### Cell migration and invasion assay

Cells were serum-starved for 24 h, then 5 × 10^4^ cells were seeded in the upper chamber (8 μm pore size, Transwell Chambers with a track-etched membrane (Corning, Inc).and Matrigel™ Invasion Chamber (BD Bioscience)) with serum-free medium, while complete medium was in the bottom chamber. Forty-eight hours later, the cells were migrated into the lower chamber and stained with 0.1% crystal violet. Migrated cells from five fields in each sample were counted for quantitative analysis. by using 24-well, New York, CA, USA).

A cell invasion assay was also conducted in the same manner but with Matrigel™ Invasion Chamber 24-well plates with 8.0-μm pores (BD Biosciences, San Jose, USA) and an incubation time of 48 h.

### Cell cycle analysis

Cells were grown to 90% confluency and then transiently transfected with control vector, CMV-miR-221/222 sponge, or T-VISA-miR-221/222 sponge. After 72 h transfection, the cells stained with propidium iodide were collected for cell cycle analysis using the BD FACS Canto II Flow Cytometer (BD Biosciences). Adhesive cells were eliminated by the scatter plot of cell fluorescence signal height and area on the fluorescence channel.

### Three-dimensional culture model

Engelbreth-Holm-Swarm extracellular matrix extract (EHS) (Matrigel, BD Biosciences) was thawed at 4 °C overnight. Prechilled culture dishes were pre-coated with a thin layer of EHS. The cells trypsinized were resuspended in cell culture medium (0.2 × 10^5^ cells/cm^2^) containing 10% EHS and poured onto the surface of the pre-coated dishes. Fresh culture medium was added into dishes every 2 to 3 days.

### Statistical analysis

One-way ANOVA analysis and t-test were used to determine statistical significance. *p* < 0.05 was considered statistically significant.

## Supplementary Information

**Additional file 1 Supplementary Table 1.** Sequence of mir221/222 inhibitor. **Supplementary Table 2.** List of antibodies for western blotting. **Supplementary Table 3.** Sequence of miR-221/222 sponge. **Supplementary Table 4.** Sequence of primers. **Supplementary Figure 1.** The morphological changes of MCF-7^TamR^ cells after the transfection with the miR-221/222 sponge expression vector.

## Data Availability

The data or plasmids generated during and/or analyzed during the current study are available from the corresponding author on reasonable request.

## Abbreviations

TamR: Tamoxifen resistance
ER: Estrogen receptor
ER^+^: ER-positive
miRs: microRNAs
3’UTR: 3′-untranslated region
MREs: miRNA response elements
CMV: Cytomegalovirus
hTERT: Human telomerase reverse transcriptase
MBSs: Multi-antisense binding sites
WPRE: The post-transcriptional regulatory element of the woodchuck hepatitis virus
