# Supplementary material for: miR-221/222 sponge abrogates tamoxifen resistance in ER-positive breast cancer cells through restoring the expression of ERα
Source: Mol Biomed. 2021 Jun 30;2:20. doi: 10.1186/s43556-021-00045-0 (PMC8607419; doi:10.1186/s43556-021-00045-0)
Supplement: Supplementary file 1 — Additional file 1 Supplementary Table 1. Sequence of mir221/222 inhibitor. Supplementary Table 2. List of antibodies for western blotting. Supplementary Table 3. Sequence of miR-221/222 sponge. Supplementary Table 4. Sequence of primers. Supplementary Figure 1. The morphological changes of MCF-7TamR cells after the transfection with the miR-221/222 sponge expression vector. [file 43556_2021_45_MOESM1_ESM.docx]

**Supplementary materials**

**Supplementary Table 1. Sequence of mir221/222 inhibitor**

| sequence | 5’ to 3’ |
| --- | --- |
| microRNA-221 inhibitor | CAAACCCAGCAGACAAUGUAGCU |
| microRNA-222inhibitor | ACCCAGUAGCCAGAUGUAGCU |
| microRNA inhibitor control | CAGUACUUUUGUGUAGUACAA |

**Supplementary Table 2. List of antibodies for western blotting**

| Antibody | Company | mono/poly | kDa |
| --- | --- | --- | --- |
| ER-α | CST, #D6R2W | Rabbit/mono | 67 |
| E-cadherin | Santa cruz, #3195T | Rabbit/mono | 135 |
| Vimentin | CST, #5741S | Rabbit/mono | 57 |
| PTEN | Santa cruz, #9559T | Rabbit/mono | 26 |
| GAPDH | Santa cruz, #sc-32233 | Mouse/mono | 42 |

**Supplementary Table 3. Sequence of miR-221/222 sponge**

| sequence | 5’ to 3’ |
| --- | --- |
| miR-221/222 sponge | ATTCTACGGGATCCGAAACCCAGCAAGGATGTAGCTCCCGAAACCCAGCAAGGATGTAGCTCCCGAAACCCAGCAAGGATGTAGCTCCCGAAACCCAGCAAGGATGTAGCTCCCACCCAGTAGAGGATGTAGCTCCCCACCCAGTAGAGGATGTAGCTCCCCACCCAGTAGAGGATGTAGCTCCCCACCCAGTAGAGGATGTAGCTCCCCACCCAGTAGAGGATGTAGCTACGCGTGCTAGCCCTCGACAATCAACCTCTGGATTACAAAATTTGTGAAAGATTGACTGGTATTCTTAACTAT |

**Supplementary Table 4. Sequence of primers**

|  | sequence | 5’ to 3’ |
| --- | --- | --- |
| miR‑221 | Forward | ACACTCCAGCTGGGAGCTACATTGTCTGCTG |
|  | Reverse | TGGTGTCGTGGAGTCG |
|  | Stem-loop | CTCAACTGGTGTCGTGGAGTCGGCAATTCAGTTGAGGAAACCCA |
| miR‑222 | Forward | ACACTCCAGCTGGGAGCTACATCTGGCTA |
|  | Reverse | TGGTGTCGTGGAGTCG |
|  | Stem-loop | CTCAACTGGTGTCGTGGAGTCGGCAATTCAGTTGAGACCCAGTA |
| U6 | Forward | CTCGCTTCGGCAGCACA |
|  | Reverse | AACGCTTCACGAATTTGCGT |
|  | Stem-loop | GTCGTATCCAGTGCAGGGTCCGAGGTATTCGCACTGGATACGACAAAATA |
| ER-α | Forward | TGCTTCAGGCTACCATTATGGA |
|  | Reverse | TGGCTGGACACATATAGTCGTT |
| PTEN | Forward | AATGGCTAAGTGAAGATGACAATCAT |
|  | Reverse | TGCACATATCATTACACCAGTTCGT |
| E-cadherin | Forward | AAAGGCCCATTTCCTAAAAACCT |
|  | Reverse | TGCGTTCTCTATCCAGAGGCT |
| Vimentin | Forward | GACGCCATCAACACCGAGTT |
|  | Reverse | CTTTGTCGTTGGTTAGCTGGT |
| β-actin | Forward | AGCGAGCATCCCCCAAAGTT |
|  | Reverse | GGGCACGAAGGCTCATCATT |


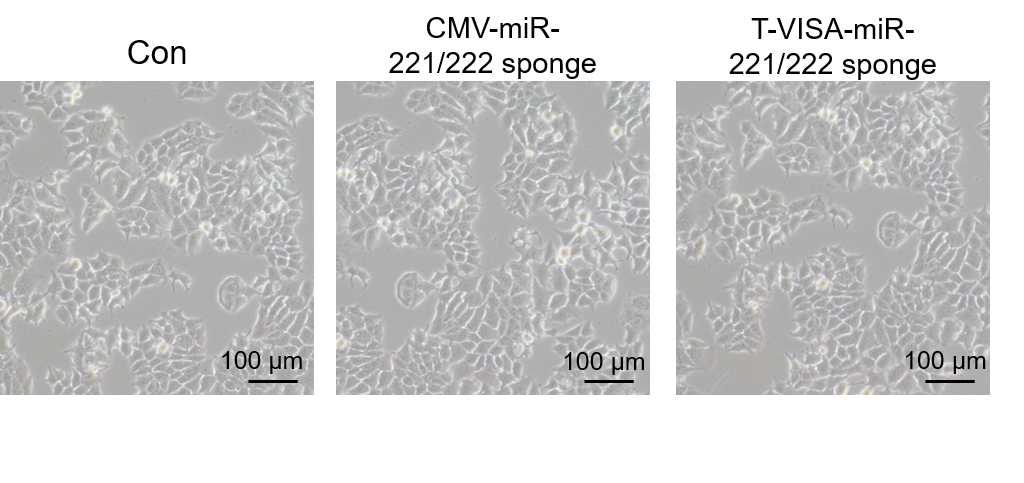


**Supplementary Figure 1.** The morphological changes of MCF-7^TamR^ cells after the transfection with the miR-221/222 sponge expression vector
